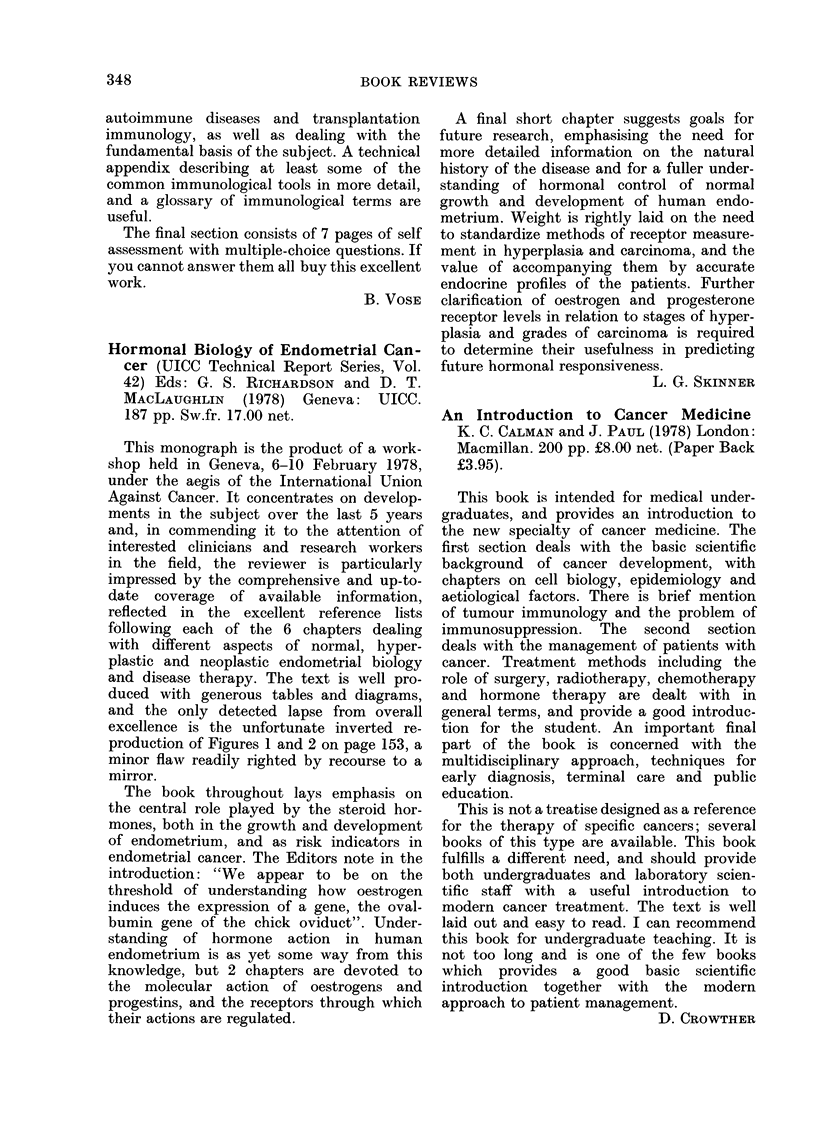# Hormonal Biology of Endometrial Cancer

**Published:** 1979-03

**Authors:** L. G. Skinner


**Hormonal Biology of Endometrial Cancer** (UICC Technical Report Series, Vol. 42) Eds: G. S. Richardson and D. T. MacLaughlin (1978) Geneva: UICC. 187 pp. Sw.fr. 17.00 net.

This monograph is the product of a workshop held in Geneva, 6–10 February 1978, under the aegis of the International Union Against Cancer. It concentrates on developments in the subject over the last 5 years and, in commending it to the attention of interested clinicians and research workers in the field, the reviewer is particularly impressed by the comprehensive and up-to-date coverage of available information, reflected in the excellent reference lists following each of the 6 chapters dealing with different aspects of normal, hyperplastic and neoplastic endometrial biology and disease therapy. The text is well produced with generous tables and diagrams, and the only detected lapse from overall excellence is the unfortunate inverted reproduction of Figures 1 and 2 on page 153, a minor flaw readily righted by recourse to a mirror.

The book throughout lays emphasis on the central role played by the steroid hormones, both in the growth and development of endometrium, and as risk indicators in endometrial cancer. The Editors note in the introduction: "We appear to be on the threshold of understanding how oestrogen induces the expression of a gene, the ovalbumin gene of the chick oviduct". Understanding of hormone action in human endometrium is as yet some way from this knowledge, but 2 chapters are devoted to the molecular action of oestrogens and progestins, and the receptors through which their actions are regulated.

A final short chapter suggests goals for future research, emphasising the need for more detailed information on the natural history of the disease and for a fuller understanding of hormonal control of normal growth and development of human endometrium. Weight is rightly laid on the need to standardize methods of receptor measurement in hyperplasia and carcinoma, and the value of accompanying them by accurate endocrine profiles of the patients. Further clarification of oestrogen and progesterone receptor levels in relation to stages of hyperplasia and grades of carcinoma is required to determine their usefulness in predicting future hormonal responsiveness.

L. G. Skinner